# Melatonin Inhibits Oxidative Stress and Apoptosis in Cryopreserved Ovarian Tissues via Nrf2/HO-1 Signaling Pathway

**DOI:** 10.3389/fmolb.2020.00163

**Published:** 2020-07-29

**Authors:** Tie Cheng Sun, Xiao Chao Liu, Song He Yang, Ling Li Song, Shan Jie Zhou, Shou Long Deng, Li Tian, Lu Yang Cheng

**Affiliations:** ^1^Faculty of Graduate Studies, Chengde Medical University, Chengde, China; ^2^Department of Immunology, Basic Medical Institute, Chengde Medical University, Chengde, China; ^3^Reproductive Medicine Center, Department of Obstetrics and Gynecology, Peking University International Hospital, Beijing, China; ^4^CAS Key Laboratory of Genome Sciences and Information, Beijing Institute of Genomics, Chinese Academy of Sciences, Beijing, China

**Keywords:** melatonin, ROS, Nrf2 signaling pathway, oxidative stress, cryopreserved ovarian tissue

## Abstract

In the field of assisted reproductive technology, female fertility preservation, particularly ovarian tissue cryopreservation in adolescent cancer patients, has attracted much attention. Melatonin (MLT) is well known for its antioxidative and anti-apoptotic properties; however, whether it can ameliorate the cryoinjury and inhibit the generation of reactive oxygen species (ROS) in cryopreserved ovarian tissues (OTs) has not yet been reported. Here, we demonstrated that MLT could protect follicular integrity; prevent cell apoptosis; decrease ROS, malondialdehyde (MDA), and nitric oxide (NO) levels; and increase activities of glutathione peroxidases (GSH-Px), glutathione (GSH), catalase (CAT), and superoxide dismutase (SOD) in cryopreserved OTs. Furthermore, these effects may be related with the activation of the nuclear factor erythroid 2-related factor 2 (Nrf2) signaling pathway, as evidenced by increased mRNA levels of Nrf2 downstream genes, including heme oxygenase-1 (HO-1), glutathione S-transferase M1 (GSTM1), SOD, and CAT. In summary, MLT can not only directly scavenge ROS but also significantly induce the activation of antioxidative enzymes via the Nrf2 signaling pathway, which is a new mechanism underlying the protection effects of MLT on cryopreserved OTs.

## Introduction

Nowadays, female fertility preservation still remains a challenge, particularly in the case of some cancer patients (Grynberg et al., 2018). Especially for prepubertal girls with cancer, whose chemotherapy and radiotherapy cannot be delayed, cryopreservation of OTs seems to be the only fertility preservation treatment modality (Van Den Heuvel-Eibrink et al., 2018; Pang et al., 2020). The development of new cryopreservation technology for tissues brings hope and enlightenment to the field of female fertility preservation. To date, 86 successful births and 9 ongoing pregnancies have been reported in women using frozen-thawed OTs worldwide (Anderson et al., 2017; Donnez and Dolmans, 2017; Jensen et al., 2017).

Cryopreservation of OTs results in physical and chemical stress, which causes morphological changes and damages phospholipid membrane, and mitochondria of follicles (Rajabi et al., 2018). Moreover, cryopreservation induces ROS generation that finally leads to oxidative stress, which affects the recovery efficiency and clinical pregnancy rate (Agarwal et al., 2012; Won et al., 2013; Grynberg et al., 2018; Van Den Heuvel-Eibrink et al., 2018; Pang et al., 2020). The production of ROS by oocytes and somatic cells and the reduced levels of antioxidant enzymes in the follicular microenvironment may induce follicular apoptosis (Gao et al., 2020; Yang et al., 2020). Some antioxidants (such as MLT and resveratrol) have been shown to counteract the negative effects of cryopreservation on the quantity and quality of OTs, probably through the enhancement of free radical scavenging mechanisms in the follicles (Tarín et al., 2002; Rocha et al., 2018; Olcese, 2020). Therefore, antioxidants are required for elimination of ROS (Tamura et al., 2020).

Melatonin (N-acetyl-5-methoxytryptamine), one of the most well-studied molecules in recent years, is mainly synthesized and secreted by pineal organ of animal and human. It acts as an antioxidant in several reproductive organs and can directly scavenge a variety of free radicals (Reiter et al., 2016; Tamura et al., 2020). It also functions to upregulate antioxidant protein expression and to protect against oxidant-mediated damage in many cryopreserved sperm/tissues (human sperm and ovary) (Li et al., 2009; Wang et al., 2012; Gao et al., 2016; Deng et al., 2017; Goodarzi et al., 2017). It is reported that oxidative stress is one of the most frequent side effects that induce follicular morphological changes and cell apoptosis in cryopreserved OTs (Goodarzi et al., 2017). Some studies demonstrated that MLT, as a highly lipophilic molecule, can easily cross the cell membranes and show antioxidant and anti-apoptotic effects (Kleszczyński et al., 2016; Reiter et al., 2016; Zhang et al., 2019; Tamura et al., 2020). Moreover, some recent studies have reported the beneficial effects of MLT on improving the preservation of OTs and auto-transplantation (Abir et al., 2017; Goodarzi et al., 2017; Gül et al., 2018). Nevertheless, the specific mechanisms of MLT underlying its protective role in stress-induced oxidative damage of follicles are less studied.

In this study, the effect and mechanism of MLT on follicles from cryopreserved OTs were investigated. We analyzed (a) follicle integrity, apoptotic rate, and antioxidant enzyme activities and (b) whether MLT can prevent the ROS production by activating the nuclear factor E2-related factor 2 (Nrf2) signaling pathway and its downstream antioxidant enzymes such as HO-1, SOD, CAT, and GSTM1.

## Materials and Methods

### Reagents

All chemical reagents used in this study were purchased from Sigma-Aldrich (St. Louis, MO, United States) unless otherwise stated.

### Animals and Experimental Design

Forty healthy female adolescent Sprague–Dawley (SD) rats (Certificate No. 11401300084289) were used. Ovaries (*n* = 80) were collected from these rats aseptically. The experimental protocols were approved by the Animal Ethics Committee of the Chengde Medical University (Chengde, China). Immediately after collection, the whole ovaries were washed with 70% alcohol for 10 s, washed twice in PBS, and kept at 4°C (Rocha et al., 2018).

Melatonin powder was dissolved in dimethyl sulfoxide (DMSO) at different concentrations and then further diluted 1:10 with vitrification solution-1 (SV-1) and vitrification solution-2 (SV-2) to yield a final concentration. The ovarian cortex was cut into approximately 3 × 3 × 1-mm fragments, randomly divided to six groups, and vitrified using different MLT concentrations [I: fresh control (*n* = 5), ovarian fragments immediately fixed; II: 0 mM MLT-untreated group (*n* = 15); III: 0.001 mM MLT-treated group (*n* = 15); IV: 0.01 mM MLT-treated group (*n* = 15); V: 0.1 mM MLT-treated group (*n* = 15); VI: 1 mM MLT-treated group (*n* = 15)]. After the freeze-thaw process, the ovarian fragments were subjected to histological analysis and TUNEL (TdT-mediated dUTP nick-end labeling) (*n* = 25), Western blotting (*n* = 25), and ROS level (*n* = 25) analysis. Each experiment was repeated at least three times (Chaves et al., 2008).

### Vitrification and Warming Procedures

The compositions and exposure times of all cryopreserved OTs are presented in Table 1. The vitrification and warming protocols were based on the previous study (Rocha et al., 2018). At the first step, the fragments were equilibrated in SV-1 and then transferred to a SV-2. The treatment with MLT used the same solutions (SV-1 and SV-2) at 0.001-mM, 0.01-mM, 0.1-mM, and 1-mM concentrations. Ovarian fragments were immersed in SV-1 for 4 min and then to SV-2 for 1 min. All the procedures were carried out at room temperature. At last, the ovarian fragments were placed in a cryopreservation tube with SV-2, exposed to nitrogen vapor for 30–60 min (−30°C), and then directly stored in liquid nitrogen (−196°C).

**TABLE 1:** Vitrification and thawing protocols.

Solutions	Compositions	Times
**Vitrification protocols**		
SV-1	TCM199 + 10 mg/mL BSA + 0.25 M sucrose + 10% EG + 10% DMSO	4 min
SV-2	TCM199 + 10 mg/mL BSA + 0.25 M sucrose + 20% EG + 20% DMSO	1 min
**Warming protocols:**		
WS-1	TCM199 + 3 mg/mL BSA + 0.5 M sucrose	5 min
WS-2	TCM199 + 3 mg/mL BSA + 0.25 M sucrose	5 min
WS-3	TCM199 + 3 mg/mL BSA	5 min
BM	PBS + 20% FBS	1–12 h

*SV-1, vitrification solutions-1; SV-2, vitrification solutions-2; WS, washing solution; BM, base medium; BSA, bovine serum albumin; EG, ethylene glycol; DMSO, dimethyl sulfoxide; FBS, fetal bovine serum.*

After 2 weeks of storage, all samples were thawed as described. The OTs were rapidly immersed in a water bath at 38°C for 1–2 min. The OTs were subjected to removal of SV-2 by using washing solutions (WS-1, WS-2, and WS-3) (Rocha et al., 2018). All OTs were kept in each solution for 5 min and then incubated for 1–12 h in a culture dish containing a base medium supplemented with PBS and 20% FBS (Won et al., 2013).

### Histological Analysis

All freeze-thawed OTs were recovered and fixed in 4% paraformaldehyde for 1 h, embedded in a paraffin block, and serially sectioned at a thickness of 4–5 μm. The sections were stained with hematoxylin–eosin, mounted, and observed under light microscopy (Olympus, Tokyo, Japan) at ×400 magnification.

Each preantral follicle type was classified according to the developmental stage (Chaves et al., 2008; Won et al., 2013):

a) Primordial follicle: oocyte surrounded by a single layer of flattened pre-granulosa cells.
b) Primary follicle: single layer of granulosa cells and oocyte surrounded by one layer of cuboidal cells.
c) Secondary follicle: oocyte surrounded by two or more layers of cuboidal granulosa cells.
d) Antral follicle: multiple layers of cuboidal granulosa cells.

The follicles were evaluated with the following morphological criteria (Gandolfi et al., 2006):

a) integrity of each follicle with oocyte and granulosa cells.
b) the presence or absence of pyknotic bodies.
c) cytoplasmic retraction.
d) the organization of granulosa cells.

Based on this evaluation, when the oocytes with non-pyknotic nuclei were surrounded by granulosa cells organized in discrete layers, they were classified as morphologically normal follicles. Degenerated follicles (abnormal) were defined as those with retracted cytoplasm, pyknotic nucleus, or disorganized granular cells detached from the basal membrane (Wang et al., 2001; Won et al., 2013). The diameters of follicles and oocytes were measured and calculated based on the average of two perpendicular axes of each morphological integrity of follicles. The ratio of morphologically intact follicles (%) was expressed as follows: the number of morphological integrity of follicles and oocytes/the total number of follicles and oocytes × 100%.

### Analysis of Apoptosis by TUNEL Assays

Cell apoptosis was analyzed by using TUNEL kit (Roche Diagnostics, Mannheim, Germany). Apoptotic follicular and oocytes were indicated by brown-yellow granules in the cytoplasm. The number of apoptotic follicles and oocytes in random fields of view (magnification, ×1000) was calculated. The apoptosis rate was expressed as follows: the number of apoptotic follicles and oocytes/the total number of follicles and oocytes × 100%.

### Western Blot Analysis

Frozen ovarian fragments were thawed and mixed with cracking liquid and centrifuged at 12,000 rpm at 4°C. Proteins in the liquid supernatant were quantitated via BCA Protein Assay Kit (Beijing Solarbio Science & Technology Co., Ltd., China). SDS-PAGE (12% protein gels) protein electrophoresis was performed, and then proteins were transferred to a PVDF membrane. After blocking with 5% non-fat milk for 60 min at room temperature, the membrane was incubated overnight at 4°C with 0.2 μg/mL rabbit anti-Bcl-2 antibody (ab59348), rabbit anti-Bax antibody (ab53154) (Abcam), and rabbit anti-GAPDH polyclonal antibody. After washing, the samples were incubated for 120 min with goat anti-rabbit IgG H&L (HRP) (ab205718). Proteins were visualized using Ultra ECL Kit [MultiSciences (LIANKE) BIOTECH, CO., LTD., Hangzhou, China] and exposed with photographic film. The images were analyzed with BandScan 5.0 software. The ratios of the gray values of the target protein to those of GAPDH were calculated as the relative level of target protein.

### Total RNA Isolation and First-Strand cDNA Synthesis

Total RNA was extracted from cryopreserved OTs using TRIzol reagent (Invitrogen, Carlsbad, CA, United States) as described previously (Kleszczyński et al., 2016). The yield of total RNA for each sample was quantified with a NanoDrop ND-1000 spectrophotometer (Thermo Fisher Scientific, Wilmington, DE, United States). First-strand cDNA of the total RNA was synthesized by reverse transcription using the SuperScript^TM^ III First-Strand Synthesis System Kit (Invitrogen, Carlsbad, CA, United States).

### Quantitative Real-Time PCR Based on Gene Expression Analysis

Quantitative real-time PCR was performed for the detection of *Nrf2*, *HO-1*, *SOD*, *CAT*, *GSTM1*, *Bcl-2*, and *Bax* mRNA expression using the LightCycler 480 II System (Roche, Mannheim, Germany) and Maxima SYBR Green qPCR Master Mix (Fermentas GmbH, St. Leon-Rot, Germany). The primers in this study are described in Table 2, and actin was used as the reference. The cDNA (10 ng) was used as a template for each sample. The amplification conditions were set as follows: 95°C for 10 min, followed by 50 cycles of denaturation at 95°C for 15 s, annealing for 30 s at 59°C, and extension for 30 s at 72°C. Relative expression of the genes was calculated with the 2-ddCt method.

**TABLE 2:** List of quantitative real-time PCR primers used in the study.

Gene	Forward primer	Reverse primer
Nrf2	5′-GGACATGGAGCAAGTTTGGC	5′-CAGCGGTAGTATCAGCCAGC
HO-1	5′-CAGGCAATGGCCTAAACTTC	5′-GCTGCCACATTAGGGTGTCT
Bcl-2	5′-GAACTGGGGGAGGATTGTGG	5′- ACCTACCCAGCCTCCGTTAT
Bax	5′-GTGAGCGGCTGCTTGTCT	5′-GAGGACTCCAGCCACAAAGA
SOD	5′-ATGGCAAAGGGCGTTGCTGTACTT	5′-TCATCCTTGAAGGCCAATAATACCA
CAT	5′-ACCCCTCCTGGACTTTTTACATC	5′-GGGATGAGAGGGTAGTCCTTGTG
GSTM1	5′-GGACTTTCCCAATCTGCCCT	5′-CTCCAAAATGTCCACACGAATCT
ACTIN	5′-GTGCTATGTTGCTCTAGACTTCG	5′-ATGCCACAGGATTCCATACC

### Measurement of ROS Generation and Antioxidant Enzyme Activities

Ovarian fragments were prepared as 10% tissue homogenates in normal saline and centrifuged at 3000 rpm at 4°C for 15 min. The supernatant was collected. The levels of ROS, GSH-Px, SOD, GSH, and CAT, as well as MDA, NO, and total antioxidant capacity (T-AOC) in OTs were measured with antioxidant kits (Nanjing Jiancheng Bioengineering Institute). Absorbance at the corresponding wavelength was determined by a microplate reader (Deng et al., 2017; Rocha et al., 2018;Yu et al., 2019).

### Cytotoxicity Assessment

The lactate dehydrogenase (LDH) released from cells to the culture medium was measured by Cytotoxicity Detection Kit (LDH) (Nanjing Jiancheng Bioengineering Institute, Nanjing, China) and evaluated with ELISA reader at 492 nm following the manufacturer’s instructions.

### Statistical Analysis

All statistical analyses were performed using SPSS version 22.0 (IBM Inc., Armonk, NY, United States). Data were expressed as mean (±standard error of the mean) of three independent experiments and presented with tables and bar graphs. The differences among the groups were analyzed by one-way analysis of variance (ANOVA), and results were considered significant when *P* < 0.05.

## Results

### Morphological Analysis of the MLT-Treated and Cryopreserved OTs

**FIGURE 1:**
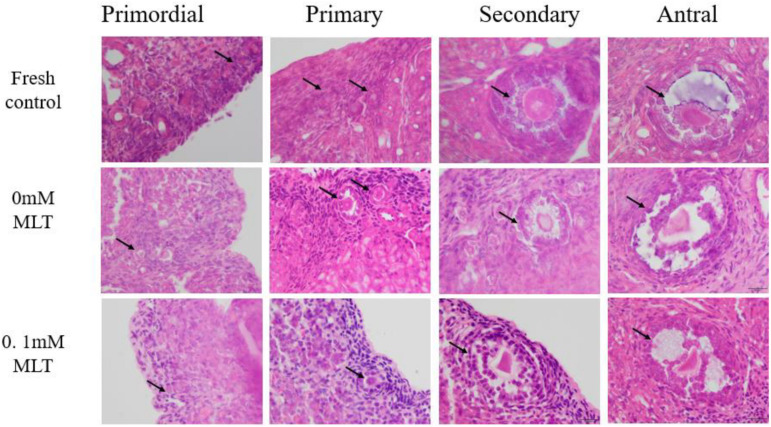
MLT protected the morphological integrity of follicles at various developmental stages in cryopreserved OTs. The horizontal panel represents the developmental stage of follicles. The vertical panel represents three groups (fresh, 0 mM, and 0.1 mM), respectively.

**FIGURE 2:**
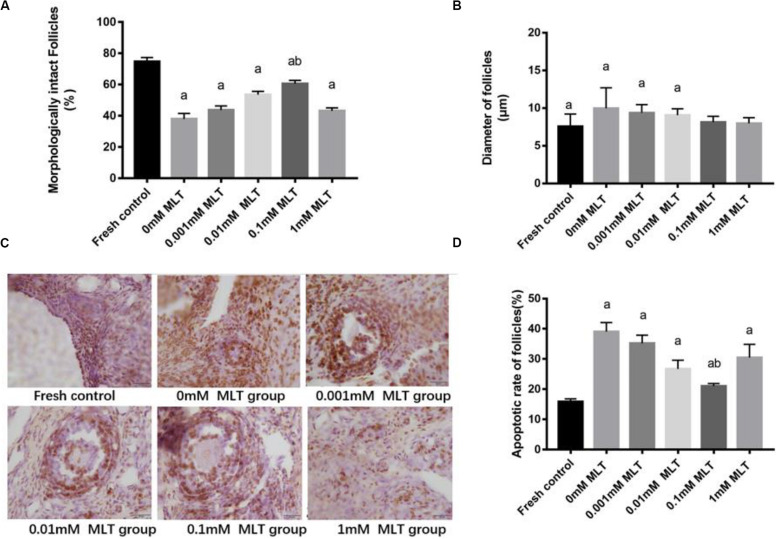
MLT protected the morphological integrity, alleviated the diameter change of follicles, and reduced the apoptotic rate in cryopreserved OTs. **(A)** Percentage of morphologically intact follicles in fresh control and each MLT-(un)treated group. **(B)** Diameter of follicles (μm) in fresh control and each MLT-(un)treated group. **(C)** Morphological integrity of follicles in fresh control and each MLT-(un)treated group. **(D)** Percentage of apoptotic rate in each MLT-(un)treated group. Data are shown as the mean ± SEM. ^a^ *P* < 0.05 compared with the 0-mM MLT-untreated group; ^b^ *P* < 0.05 compared with the MLT-treated group.

The procedure of freeze-thaw OTs will produce a large number of ROS, cause damage to various stages of developmental follicles, and ultimately compromise the outcome of OT transplantation and pregnancy rate. The morphological changes were measured in vitrified OTs. The morphology of follicles and oocytes was irregular and deformed, with granular cells arranged loosely and detached from the stromal membrane (Figure 1). In all of the five groups, the number of follicles at various stages with morphological integrity significantly decreased and the diameters of follicles and oocytes increased compared to fresh OTs (Figures 2A–C). However, the percentages of morphologically intact follicles in MLT-treated groups were markedly increased by ∼40% (0.001 mM, *P* < 0.05), ∼45% (0.01 mM, *P* < 0.05), ∼65% (0.1 mM, *P* < 0.01), and ∼66% (1 mM, *P* < 0.01), respectively, compared with those in the non-MLT-treated group (Figure 2A). These results indicate that 0.1 mM and 1 mM MLT can effectively protect the follicular integrity.

In addition to protecting follicles at various stages, MLT decreased the apoptotic rates during TUNEL assays in Figure 2D. Significant decreases in apoptotic rates (*P* < 0.005) were shown in MLT-treated groups, among which 0.1 mM MLT had the optimal effect (*P* < 0.001), which was consistent with the morphological analysis results in Figures 1, 2A–C.

### Expression Levels of Bax and Bcl-2 in Cryopreserved OTs

**FIGURE 3:**
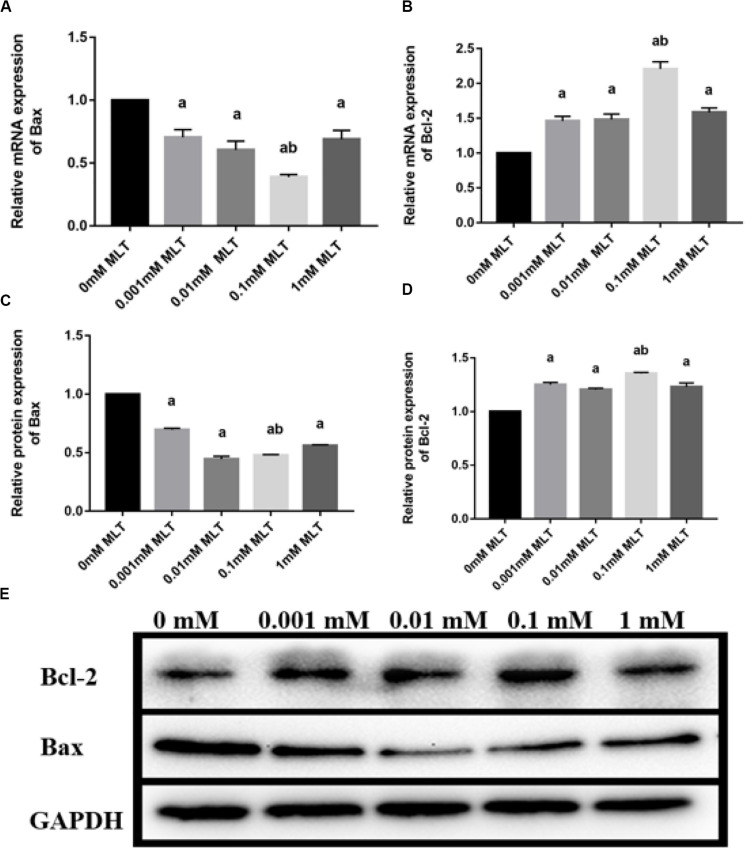
Effect of MLT on mRNA and protein expressions of Bax and Bcl-2 in cryopreserved OTs. **(A)** Relative mRNA expressions of Bax in each MLT-(un)treated group. **(B)** Relative mRNA expressions of Bcl-2 in each MLT-(un)treated group. **(C)** Protein expressions of Bax in each MLT-(un)treated group. **(D)** Protein expressions of Bcl-2 in each MLT-(un)treated group. **(E)** Representative Western blot image of Bax and Bcl-2 in each MLT-(un)treated group. Data are shown as the mean ± SEM. ^a^ *P* < 0.05 compared with the 0-mM MLT-untreated group; ^b^ *P* < 0.05 compared with the MLT-treated group.

Bax and Bcl-2 expression levels were further detected with RT-PCR and Western blot. The mRNA and protein expressions of Bax in the 0.1-mM MLT group significantly declined compared to those in the 0-mM MLT group (*P* < 0.01) and the other groups (*P* < 0.05, in Figures 3A,C,E). Figures 3B,D,E showed a statistically significant increase in Bcl-2 mRNA and protein expressions in the 0.1-mM MLT group (*P* < 0.05). These results suggest that 0.1 mM MLT has an optimal anti-apoptosis effect when MLT is used as cryoprotectant.

### Effect of MLT on Oxidative Stress in Cryopreserved OTs

**FIGURE 4:**
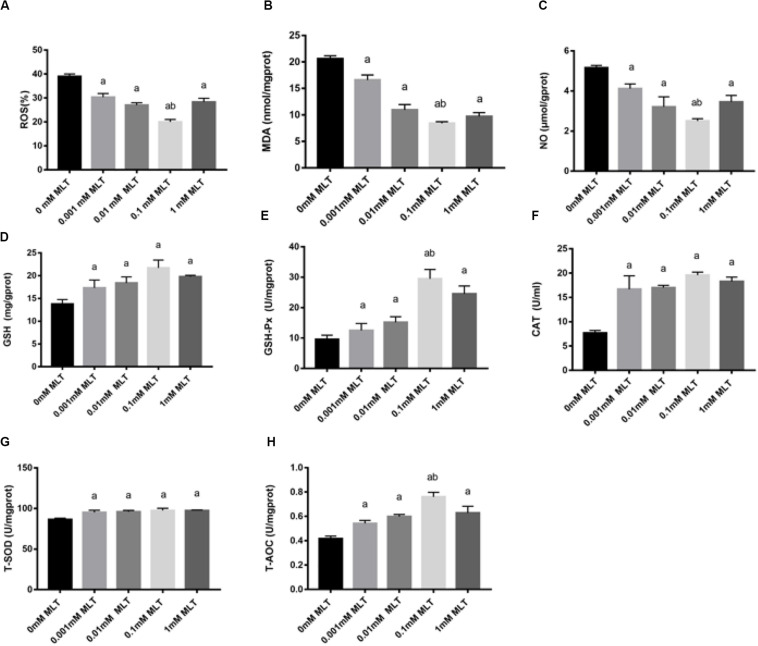
MLT alleviated oxidative stress in cryopreserved OTs. ROS **(A)**, MDA **(B)**, NO **(C)**, GSH **(D)**, GSH-Px **(E)**, CAT **(F)**, T-SOD **(G)**, and T-AOC **(H)**. Data are shown as the mean ± SEM. ^a^ *P* < 0.05 compared with the 0-mM MLT-untreated group; ^b^ *P* < 0.05 compared with the MLT-treated group.

In order to detect the oxidative damage status of cryopreserved OTs, the levels of ROS, MDA, and NO were analyzed. ROS generation was decreased after pretreatments with MLT by ∼35% (0.001 mM MLT, *P* < 0.01), ∼28% (0.01 mM MLT, *P* < 0.01), ∼18% (0.01 mM MLT, *P* < 0.001), and ∼25% (1 mM MLT, *P* < 0.01), respectively (Figure 4A). As shown in Figures 4B,C, similar results were found for the levels of MDA and NO compared with the 0-mM MLT group (*P* < 0.01). Meanwhile, consistent with the findings mentioned above, the highest antioxidant activity was observed in the 0.1-mM MLT group.

In addition, activities of GSH, GSH-Px, CAT, SOD, and T-AOC were also measured in order to detect the antioxidant capacity of MLT in cryopreserved OTs. As shown in Figures 4D–H, the activities of GSH, GSH-Px, CAT, T-SOD, and T-AOC significantly decreased in the groups treated with various concentrations of MLT (especially at the 0.1 mM), compared with those in the 0-mM MLT group (*P* < 0.01).

### MLT Promotes Nrf2 and Its Downstream Related Gene Expression in Cryopreserved OTs

**FIGURE 5:**
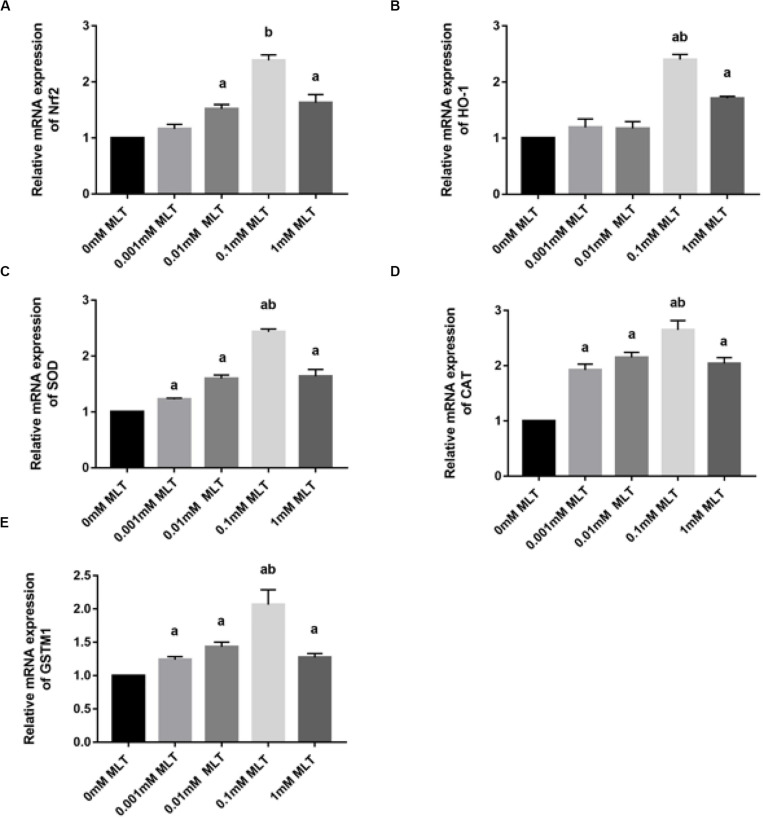
MLT affects Nrf2 and its related gene expressions in cryopreserved OTs. **(A)** Relative mRNA expressions of *Nrf2* in each MLT-(un)treated group. **(B)** Relative mRNA expressions of *HO-1* in each MLT-(un)treated group. **(C)** Relative mRNA expressions of *SOD* in each MLT-(un)treated group. **(D)** Relative mRNA expressions of *CAT* in each MLT-(un)treated group. **(E)** Relative mRNA expressions of *GSTM1* in each MLT-(un)treated group. Data are shown as the mean ± SEM. ^a^ *P* < 0.05 compared with the 0-mM MLT-untreated group; ^b^ *P* < 0.05 compared with the MLT-treated groups.

Melatonin has been well investigated as a strong antioxidant, which is involved in many activities of protection via the Nrf2 signaling pathway. To determine the effect of MLT on key genes in the Nrf2 signaling pathway, RT-PCR was performed. As shown in Figure 5A, the level of *Nrf2* mRNA in the 0.1-mM MLT group was significantly higher than those in the 0-mM MLT group (*P* < 0.01) or other groups with different concentrations of MLT (*P* < 0.001).

A similar pattern of regulation was observed at the gene expression levels of Nrf2 downstream target genes including *HO-1*, *SOD*, *CAT*, and *GSTM1* (Figures 5B–E). A striking gene upregulation was immediately observed in the MLT-treated groups (*P* < 0.001). Moreover, MLT at the dose of 0.1 mM, which was considered as the optimal MLT concentration, upregulated the gene expression of analyzed enzymes directly by ∼2.5 times (*HO-1* and *SO*D, *P* < 0.001, Figures 5B,C) and ∼1.5 times (*CA*T *P* < 0.01, Figure 5D). However, the expression surge of *GSTM1* mRNA was not as obvious as that of other Nrf2 downstream genes, although it also showed a higher level at the concentration of 1 mM MLT than other groups (Figure 5E).

### MLT Cytotoxic Analysis

**FIGURE 6:**
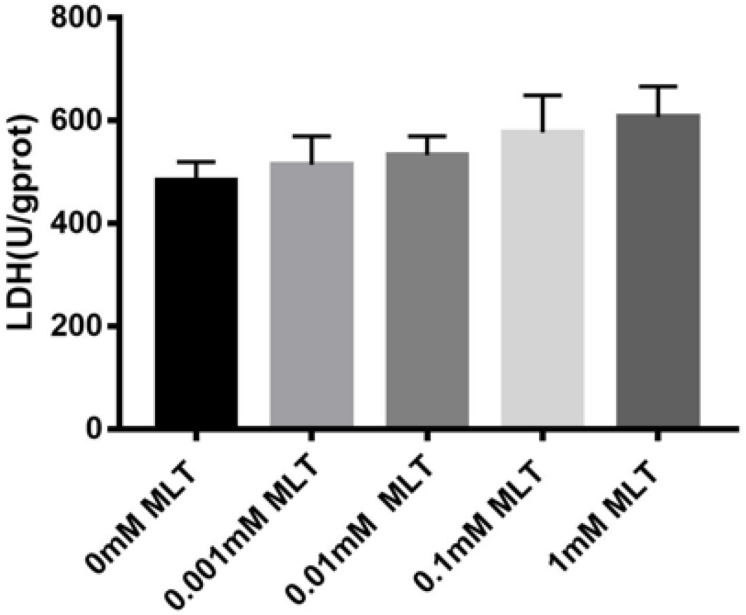
Lactate dehydrogenase (LDH) assay. MLT in the vitrification medium at the indicated concentrations is not cytotoxic. Data are shown as the mean ± SEM.

Safety, especially no cytotoxicity, is always one of the biggest concerns of all kinds of cryoprotectants. In order to verify whether MLT can produce a cytotoxic effect on OTs as a cryoprotective additive, the content of LDH was measured. Although the content of LDH increased slightly after adding MLT, there were no significant differences between MTL-treated groups (*P* > 0.05) (Figure 6).

## Discussion and Conclusion

This study is to evaluate the protective role of MLT at different concentrations in cryopreservation of OTs. Morphological changes and apoptotic rates in MLT-treated groups were compared after vitrification. We found that the 0.1-mM MLT-treated and 1-mM MLT-treated groups retained follicular morphological integrity most effectively after vitrification. Only the OTs vitrified with 0.1 mM MLT maintained a high percentage of morphological integrity comparable to fresh control. Our findings are consistent with previous reports showing that MLT-vitrificated solutions can protect the follicular morphological integrity in cryopreserved OTs and might help manage the negative effects of cryopreservation on female fertility preservation (Abir et al., 2017; Goodarzi et al., 2017).

This research found that the freeze-thaw process induced an increased apoptotic rate of follicular cells, which is in line with previous studies (Chaves et al., 2008; Won et al., 2013; Rocha et al., 2018). Studies have demonstrated the anti-apoptotic effects of some cryoprotectants, such as resveratrol and cryoprotective agent after vitrification of OTs (Won et al., 2013; Rocha et al., 2018). Notably, MLT can attenuate the stress-induced apoptotic follicular cell damage in the ovary (Barberino et al., 2017; Jang et al., 2017). Similarly, our results also showed that the numbers of intact follicles and apoptotic follicular cells were significantly reduced by MLT during the freeze-thaw processes. We also detected the level of apoptosis-related proteins in cryopreserved OTs and found that there was a decreased level of Bax (pro-apoptotic protein) and an increased level of Bcl-2 (anti-apoptotic protein), especially in the 0.1-mM MLT-treated group. Therefore, these findings above strongly support that MLT has an ideal anti-apoptotic effect.

Oxidative stress is one of the main factors that induce follicular cell apoptosis (Won et al., 2013; Barberino et al., 2017; Rocha et al., 2018; Zhang et al., 2019). MLT, as an antioxidant, has protective effects against oxidative stress and apoptosis (Reiter et al., 2016). It has also been reported that MLT, as a cryoprotectant, plays an important role in the cryopreservation of human semen (Karimfar et al., 2015; Najafi et al., 2018). However, whether MLT can protect cryopreserved OTs from oxidative damage still remains unknown.

Glutathione peroxidases catalyzes the reduction of hydrogen peroxide by GSH and thus can protect the integrity of cell membrane structure and function (Czuczejko et al., 2019). GSH is the major antioxidant sensitive to intracellular ROS (Yu et al., 2019). SOD and CAT are the first line of defense against oxidative stress by directly scavenging ROS in the defense system (Ozkan et al., 2018; Yu et al., 2019). MDA is the final product of lipid peroxidation and a biomarker indicating the level of oxidative stress. NO successively increased the oxidation stress. MLT can counteract the generation of NO by inhibiting the activity of inducible nitric oxide synthase (Mayo et al., 2005). Our results demonstrated that MLT reduced the ROS, NO, and MDA levels, meanwhile it promoted antioxidant enzyme activities (GSH, GSH-Px, T-SOD, CAT, and T-AOC), which are in line with recent studies (Deng et al., 2017; Yu et al., 2019). Therefore, MLT can not only scavenge free radicals but also promote the activity of antioxidant enzymes and prevent against oxidative stress in cryopreserved OTs.

The Nrf2 signaling pathway was further studied to investigate the protective mechanism of MLT. Nrf2 plays a significant role in regulating the expression of antioxidant genes (Lim et al., 2015; Akino et al., 2018; Khadrawy et al., 2019). In this study, MLT promoted *Nrf2* mRNA expression in cryopreserved OTs, suggesting that the activation of the Nrf2 signaling pathway may be the main mechanism of MLT in protecting the cryopreserved OTs. The mechanism of MLT activating the Nrf2 signaling pathway remains to be further investigated. HO-1 is also reported to be involved in ovarian response to oxidative stress (Bergandi et al., 2014; Yan et al., 2018; Yu et al., 2019). In this study, *HO-1* mRNA expression levels were found to be significantly higher in cryopreserved OTs. In addition, Nrf2 exerts the protective mechanism by regulating expressions of Nrf2 downstream target genes (including HO-1, CAT, SOD, and GSTM1) in cryopreserved OTs. Many studies found that MLT is a potent regulator of Nrf2 and HO-1 in oxidative damage (Kang et al., 2018; Niringiyumukiza et al., 2019). Therefore, it can be assumed that restraint stress can achieve oxidative stress by interfering with the Nrf2/HO-1 signaling pathway in cryopreserved OTs. However, these results may lack adequate demonstration on whether HO-1 is directly regulated by MLT or depends on Nrf2. This finding, while preliminary, suggests that the protective effects of MLT are at least partially attributable to normalization of Nrf2 and HO-1 expression to attenuate ROS produced by restraint stress.

**FIGURE 7:**
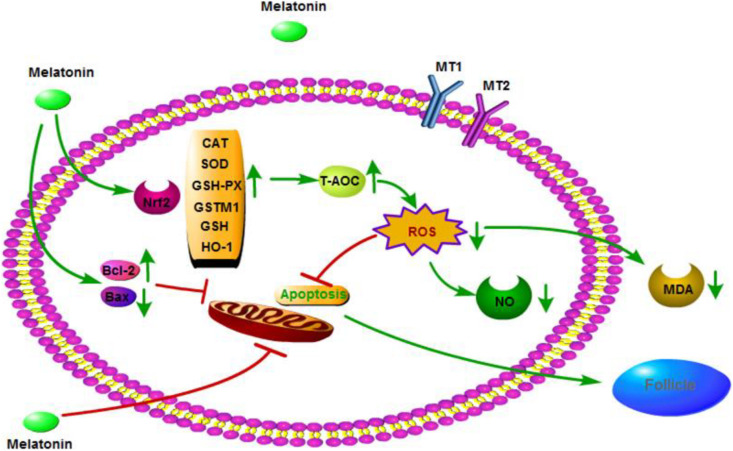
Mechanisms by which melatonin ameliorates oxidative stress via modulation of the Nrf2 pathway in cryopreserved OTs.

In conclusion, MLT may have a significant protective effect on cryopreserved OTs by inhibiting oxidative stress and apoptosis, as well as activating the Nrf2 signaling pathway (Figure 7). These results provide a new method for cryopreservation of OTs in female fertility preservation.

## Data Availability Statement

All datasets presented in this study are included in the article/supplementary material.

## Ethics Statement

The animal study was reviewed and approved by the Animal Ethics Committee of the Chengde Medical University (Chengde, China). Written informed consent was obtained from the owners for the participation of their animals in this study.

## Author Contributions

TS, SD, and LC conceived and designed the experiments. TS, XL, SY, LS, and SZ performed the experiments. TS, XL, SY, and LT analyzed the data. LC and LT contributed to reagents, materials, and analysis tools. TS and LC wrote the manuscript. All authors contributed to the article and approved the submitted version.

## Conflict of Interest

The authors declare that the research was conducted in the absence of any commercial or financial relationships that could be construed as a potential conflict of interest.

## Abbreviations

CAT: catalase
GSH: glutathione
GSH-Px: glutathione peroxidases
GSTM1: glutathione S-transferase M1
HO-1: heme oxygenase-1
MDA: malondialdehyde
MLT: melatonin
NO: nitric oxide
Nrf2: nuclear factor erythroid 2-related factor 2
OTs: ovarian tissues
ROS: reactive oxygen species
SOD: superoxide dismutase.
